# Prognostic Value and Immunological Characteristics of a Novel RNA Binding Protein Signature in Cutaneous Melanoma

**DOI:** 10.3389/fgene.2021.723796

**Published:** 2021-08-31

**Authors:** Jun Tian, Chongzhi Ma, Li Yang, Yang Sun, Yuan Zhang

**Affiliations:** ^1^Department of Dermatology, Shaanxi Provincial People’s Hospital, Xi’an, China; ^2^Department of Dermatology, The 63600 Hospital of PLA, Lanzhou, China; ^3^Department of Oncology, Shaanxi Provincial People’s Hospital, Xi’an, China

**Keywords:** cutaneous melanoma, immune cell infiltration, tumor microenvironment, prognosis, prognostic signature

## Abstract

**Background:**

The existing studies indicate that RNA binding proteins (RBPs) are closely correlated with the genesis and development of cancers. However, the role of RBPs in cutaneous melanoma remains largely unknown. Therefore, the present study aims to establish a reliable prognostic signature based on RBPs to distinguish cutaneous melanoma patients with different prognoses and investigate the immune infiltration of patients.

**Methods:**

After screening RBPs from the Cancer Genome Atlas (TCGA) and Gene Expression Omnibus (GEO) databases, Cox and least absolute shrinkage and selection operator (LASSO) regression analysis were then used to establish a prediction model. The relationship between the signature and the abundance of immune cell types, the tumor microenvironment (TME), immune-related pathways, and immune checkpoints were also analyzed.

**Results:**

In total, 7 RBPs were selected to establish the prognostic signature. Patients categorized as a high-risk group demonstrated worse overall survival (OS) rates compared to those of patients categorized as a low-risk group. The signature was validated in an independent external cohort and indicated a promising prognostic ability. Further analysis indicated that the signature wasan independent prognostic indicator in cutaneous melanoma. A nomogram combining risk score and clinicopathological features was then established to evaluate the 3- and 5-year OS in cutaneous melanoma patients. Analyses of immune infiltrating, the TME, immune checkpoint, and drug susceptibility revealed significant differences between the two groups. GSEA analysis revealed that basal cell carcinoma, notch signaling pathway, melanogenesis pathways were enriched in the high-risk group, resulting in poor OS.

**Conclusion:**

We established and validated a robust 7-RBP signature that could be a potential biomarker to predict the prognosis and immunotherapy response of cutaneous melanoma patients, which provides new insights into cutaneous melanoma immunotherapeutic strategies.

## Introduction

Cutaneous melanoma is the most aggressive and dangerous skin malignancy with high levels of morbidity, and its incidence continues to increase each year (Swetter et al., 2019). Patients with early-stage can usually be cured by surgical resection, and more than 90% of patients survive for more than 5 years (Siegel et al., 2020). Once metastasis occurs, patients suffer from a dismal prognosis with a median overall survival (OS) of only 6 to 10 months, and the 5-year survival rate is dismal (<10%) (Schadendorf and Hauschild, 2014; Tang et al., 2016). Generally, the risk stratification and prognosis of patients with melanoma are mainly determined by clinical characteristics, such as Breslow thickness, ulcers, and lymphatic vascular infiltration (Hyams et al., 2019). Nevertheless, due to the phenotype and genetic heterogeneity of malignant melanoma, conventional clinicopathological features are still limited or restricted in their ability to accurately predict individual outcomes (Diamantopoulos and Gogas, 2016). Therefore, these sobering data highlight the urgent need for the development of novel malignant melanoma-specific genomic models to accurately predict clinical outcomes of melanoma patients and provide a guide to more effective individual therapies.

RNA binding proteins (RBPs) effectively and ubiquitously regulate transcripts throughout their life cycle (Corley et al., 2020). RBPs contain a large class of more than 2,000 proteins that play vital roles in multiple RNA processes, including stability, transport and translation, splicing, and degradation of RNAs (Mohibi et al., 2019; Corley et al., 2020). Recent studies have shown that RBPs not only affect normal cell processes but also have become major players in the initiation and progression of cancer (Masuda and Kuwano, 2019; Schuschel et al., 2020; Weiße et al., 2020). Dysregulation, localization, or post-translational modification of RBPs can not only increase the expression of oncogenes but also promote tumorigenesis by reducing the expression of tumor suppressor genes. For example, the RBPs RBM38 and RBM24, as single members of the RBP family containing RRM, have similar functions by regulating the same target. Both RBM38 and RBM24 can be induced by the tumor suppressor p53, thereby inhibiting the translation of p53 mRNA (Zhang et al., 2011; Zhang M. et al., 2018). RBM38 promotes or inhibits tumor formation mainly depends on the state of p53 because RBM38 can inhibit the expression of wild-type and mutant p53 through mRNA translation (Zhang et al., 2014). PCBP1 has been reported to be a tumor suppressor to inhibit tumorigenesis, development, and metastasis in several types of cancer (Zhang et al., 2020). Elevated PCBP1 was found to promote p27 mRNA stability and translation, but inhibit the expression of oncogenic STAT3 isoform and MAPK1 (Shi et al., 2018; Zhang Y. et al., 2018; Wang et al., 2019). The RBPs hnRNP K has been reported to upregulate the expression of several oncogenes, such as MYC and Src (Adolph et al., 2007; Gallardo et al., 2020). However, the mechanisms by which most RBPs cause cancer is still unknown.

In the present study, we constructed a robust 7-RBP prognostic signature based on public datasets. The signature was verified in an independent external cohort and indicated a promising predictive ability. Then, a nomogram combining risk score and clinicopathological characteristics was then established to evaluate the 3- and 5-year OS in cutaneous melanoma patients. Analyses of immune infiltrating, immune-related pathways, TME, immune checkpoint, and drug susceptibility revealed significant differences between the two groups. GSEA analysis revealed that basal cell carcinoma, notch signaling pathway, melanogenesis pathways were enriched in the high-risk group, resulting in poor OS.

## Materials and Methods

### Data Source and Preprocessing

The RNA-sequencing profiles and clinical data for TCGA SKCM cohort were obtained from The Cancer Genome Atlas (TCGA) database^1^. SKCM-related datasets GSE65904 from the GEO database^2^ were used as an independent external validation set. For data cleaning, samples with missing clinical data were excluded. After preprocessing, there were 413 samples in the TCGA dataset, 210 in the GSE65904 dataset. The clinical statistics information is shown in Table 1. A total of 1542 genes coding for RBPs were obtained from the previous publications (Baltz et al., 2012; Castello et al., 2012; Kwon et al., 2013; Cunningham et al., 2015).

**TABLE 1:** Clinicopathologic characteristics of melanoma patients in TCGA and GEO cohorts.

Variables	TCGA cohort	GSE65904 cohort
	
	(*n* = 413)	(*n* = 210)
	N (%)	N (%)
**Age**		
≤60	218 (52.8)	83 (39.5)
>60	195 (47.2)	127 (60.5)
**Gender**		
Female	156 (37.8)	86 (41.0)
Male	257 (62.2)	124 (59.0)
**Tumor type**		
Primary tumor	99 (24.0)	/
Regional cutaneous	64 (24.0)	/
Regional lymph node	198 (47.9)	/
Distant metastasis	52 (12.6)	/
**Breslow depth**		
≤1.5	101 (24.5)	/
1.5–3.0	71 (17.2)	/
>3	165 (40.0)	/
Unknown	76 (18.4)	/
**Ulceration**		
No	135 (32.7)	/
Yes	160 (38.7)	/
Unknown	118 (28.6)	/
Stage	122 (32.5)	
I-II	218 (52.8)	/
III-IV	195 (47.2)	/
**Tumor status**		
Tumor free	164 (39.7)	/
With tumor	95 (23.0)	/
Unknown	154 (37.3)	/

### Prognostic Signature Construction

To screen the prognostic related RBPs, univariate Cox regression analysis was conducted to evaluate the relationship between the expression level of RBPs and the OS of patients in the TCGA cohort. P-value < 0.05 was set as cutoff criteria. To minimize the risk of over-fitting and remove highly related genes, the “glmnet” R package was used for Lasso regression analysis, and the stepwise multiple Cox regression method was used to establish the optimal model. Risk score = Exp1 × β1 + Exp 2 × β2 + ⋯ + Exp n × βn. β is the regression coefficient, while Exp represented gene expression level. Based on the median of estimated risk score, patients were categorized into low- and high-risk subgroups. Survival analyses were carried out for the comparison of the prognostic outcomes between two subgroups using the “survival” and “survminer” R packages. Further, the ROC curves were applied to assess the predictive capabilities of the above signature by “SurvivalROC” R package. In addition, principal component analysis (PCA) and t-SNE were carried out to explore the different gene expression patterns of the two risk groups.

### Validation of Prognostic Signature

Using the same method as that used in the training dataset, the risk score of each patient in the GSE65904 validation dataset and the corresponding median risk scores were calculated separately, after which the patients were grouped two groups (high and low). The survival curves of the two groups were plotted using the Kaplan-Meier method. Time-dependent area under the curve (AUC) analysis was performed to assess the predictive performance of the model.

### Immune Infiltrating Analysis

Given the critical role of immune infiltrating cells in cutaneous melanoma tumorigenesis and development, the abundance of 22 immune cell types were calculated by CIBERSORT^3^ algorithm (Newman et al., 2015). The tumor microenvironment (TME) scores of each single melanoma patient were estimated using the ESTIMATE algorithm (Yoshihara et al., 2013). In addition, the expression of the immune checkpoint was used to examine the molecular relationship with the prognostic signature.

### Drug Susceptibility Analysis

We use the R software package “pRRophetic” to predict the antineoplastic drug susceptibility for patients with the high- and low-risk groups. The regression analysis was conducted to obtain the half-maximal inhibitory concentration (IC50) estimated value of each specific antineoplastic drug treatment.

### Development and Validation of a Prognostic Nomogram

The univariate and multivariate Cox regression analyses were conducted to detect whether this signature can act as an independent prognostic factor for cutaneous melanoma patients. Stratification analyses were performed to further validate the predictive accuracy of the model. These variables include age (≤60 and >60 years), gender (male and female), tumor stage (I–II and III–IV), Breslow depth (≤1.5, 1.5–3.0, and >3.0), tumor type (primary tumor, regional cutaneous, regional lymph node, and distant metastasis), ulceration (yes and no), and tumor status (tumor-free and with tumor). To quantitatively estimate cutaneous melanoma prognosis in clinical practice, a prognostic nomogram that integrated both the signature, age, and tumor stage was generated based on the multivariable Cox regression analysis. The ROC curve and calibration plot were drawn to estimate the predictive performance and discriminating ability of the nomogram scoring system.

### Gene Set Enrichment Analysis (GSEA)

Gene set enrichment analysis software (version 4.1.0) was utilized to investigate the meaningful biological processes that might be involved in causing the different prognoses between low- and high-risk groups based on the Hallmarks gene collection file (C2cp.kegg.v7.2.symbols.gmt). The number of permutations was set to 1,000 times, and the “phenotype labs” were set to high-risk score versus low-risk score. The outcomes meet FDR *q* < 0.25 and NOM *p* < 0.05 were considered significant.

### Statistical Analyses

All statistical analyses were implemented using R version 4.0.4. We used the Chi-squared test and Fisher’s exact test to evaluate the differences in categorical data between different datasets and groups and the Mann-Whitney U test or Student t-test to compare the quantitative data.

## Results

### Identification of RBPs in Cutaneous Melanoma

**FIGURE 1:**
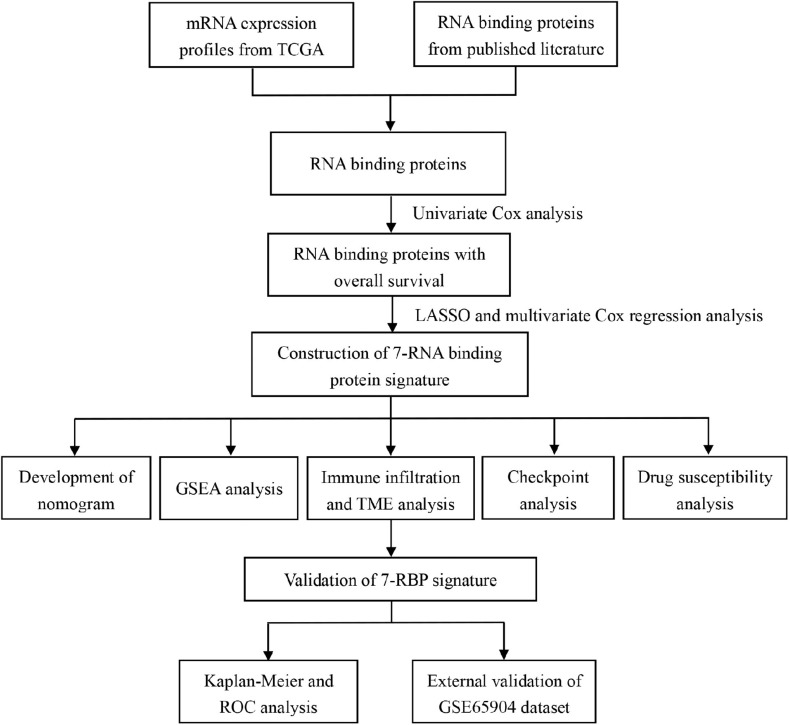
Flow diagram showing the design of the study.

Figure 1 showed the research idea about this study. A systematic analysis was carried out for the critical roles and the potential prognostic values of RBPs played in cutaneous melanoma. At first, we downloaded transcriptome information and clinical data from TCGA and GEO datasets. Then, a total of 1541 RBPs were acquired from previous publications, which were integrated with the mRNA from the TCGA database to obtain 1492 RBPs in cutaneous melanoma. A total of 1374 RBPs were identified by taking the intersection of 1492 RBPs and mRNAs from the GEO dataset.

### Construction of the RBPs-Related Signature

**FIGURE 2:**
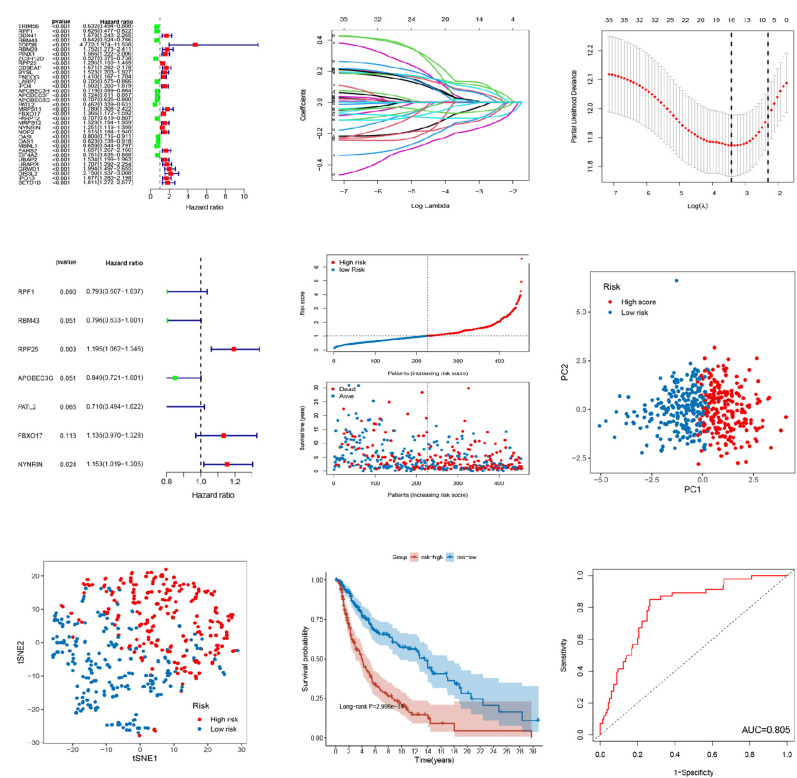
Establishment of 7-RBP signature in TCGA dataset. **(A)** Forest plot of univariate Cox regression analysis for prognostic RBPs. **(B,C)** The LASSO regression analysis and partial likelihood deviance on the prognostic RBPs. **(D)** Forest plot of multivariate cox regression analysis for prognostic RBPs. **(E)** The ranked dot plot indicates the risk score distribution and scatter plot presenting the patients’ survival status. **(F,G)** The PCA and t-SNE analyses were performed between the two risk groups. **(H)** KM analysis of the OS between the two groups. **(I)** ROC curve of 7-RBP signature.

The relationship between the expression of these 1374 RBPs and OS was analyzed by univariate Cox regression. As a result, 35 RBPs were left as prognostic-associated candidates (*P* < 0.001) (Figure 2A). Then, sixteen prognostic RBPs were conducted with LASSO regression analysis (Figure 2B) and partial likelihood deviance (Figure 2C). Subsequently, multivariate Cox regression analysis on the 16 RBPs was conducted to further select a robust and effective risk model for prognosis prediction and identified 7 RBPs (RPF1, RBM43, RPP25, APOBEC3G, PATL2, FBXO17, and NYNRIN) (Figure 2D). A prognostic signature based on 7 RBPs, including 3 high-risk RBPs (RPP25, FBXO17, and NYNRIN) and 4 low-risk RBPs (RPF1, RBM43, APOBEC3G, and PATL2), and the risk score were obtained. The risk score was obtained in line with the expression quantities of the 7 RBPs in various samples and the correlation coefficients. The risk score = (−0.23148 × level of RPF1) + (−0.22826 × level of RBM43) + (0.17848 × level of RPP25) + (−0.16324 × level of APOBEC3G) + (−0.34232 × level of PATL2) + (0.12702 × level of FBXO17) + (0.14216 × level of NYNRIN). After scoring each patient’s risk through the signature, patients above and below the mean risk score were assigned to the high- and low-risk group, respectively. Figure 2E showed the status and survival time of patients in the training set. PCA and t-SNE analyses indicated that discernible dimensions between high- and low-score patients (Figures 2F,G). Comparing the KM curves of the two groups, we found a significant difference in the OS of patients between the two groups. Patients with highrisk have an expressively lower OS than those with low-risk (*P* < 0.001, Figure 2H). The AUC of the ROC curves was 0.805 in the training cohort, suggesting the great predictive performance of this signature (Figure 2I).

### External Validation of the Prognostic Significance of RBPs

**FIGURE 3:**
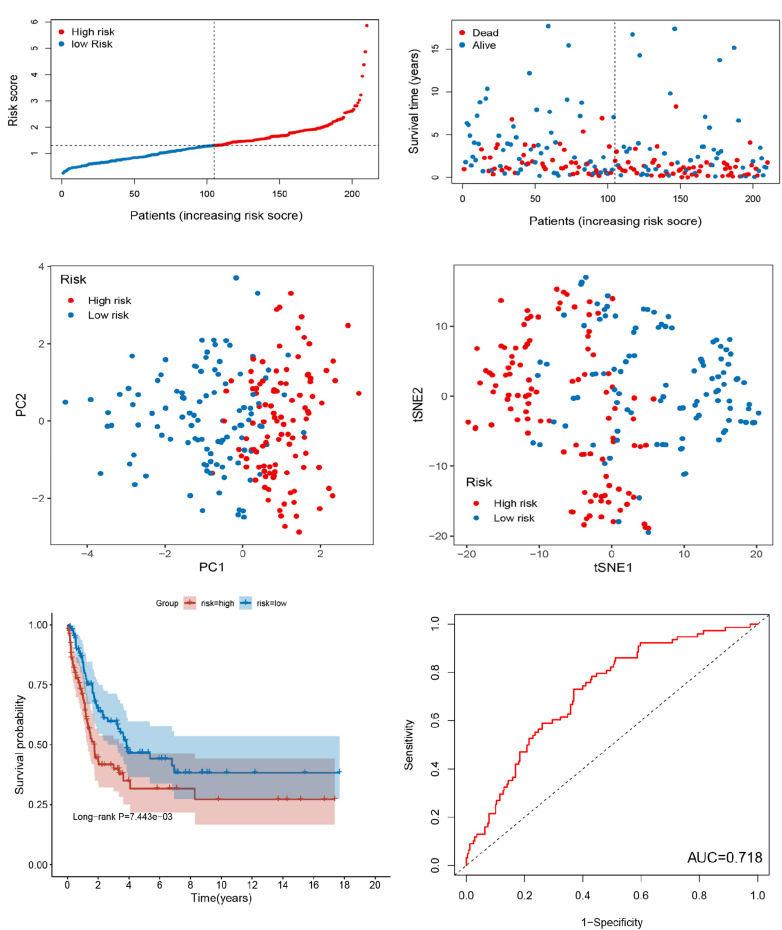
Evaluation of 7-RBP signature in GSE65904 dataset. **(A)** The ranked dot plot indicates the risk score distribution. **(B)** Scatter plot presenting the patients’ survival status. **(C,D)** The PCA and t-SNE analyses were performed between the two risk groups. **(E)** KM analysis of the OS between the two groups. **(F)** ROC curve of 7-RBP signature.

The GSE65904 dataset was separately used to determine the validity and robustness of the signature as an independent validation cohort (Figures 3A–F). The risk scores, survival status of patients, and PCA and t-SNE demonstrating the variation tendencies of high- and low-risk groups were, respectively, as shown in Figures 3C,D. As shown in Figure 3E, the OS between the high- and low-risk groups was proved to be statistically different (*P* < 0.001), which is consistent with the training set. The AUC for this risk score signature is 0.718, proving that the model has a promising predictive value (Figure 3F). To understand whether this newly identified RBP signature can specifically predict prognosis of cutaneous melanoma or has general prognostic value for other cancers, we evaluated prognostic value of the RBP signature in 32 cancer types using the TCGA pan-cancer data and found that the signature can also predict prognosis of other 5 types of cancer (Supplementary Figures 1, 2), including bladder urothelial carcinoma (TCGA-BLCA), breast invasive carcinoma (TCGA-BRCA), ovarian serous cystadenocarcinoma (TCGA-OV), mesothelioma (TCGA-MESO), and lung adenocarcinoma (TCGA-LUAD). Taken together, these results further validated that signature has high validity for survival prediction in cutaneous melanoma.

### Correlation of the Signature With TME in Cutaneous Melanoma

**FIGURE 4:**
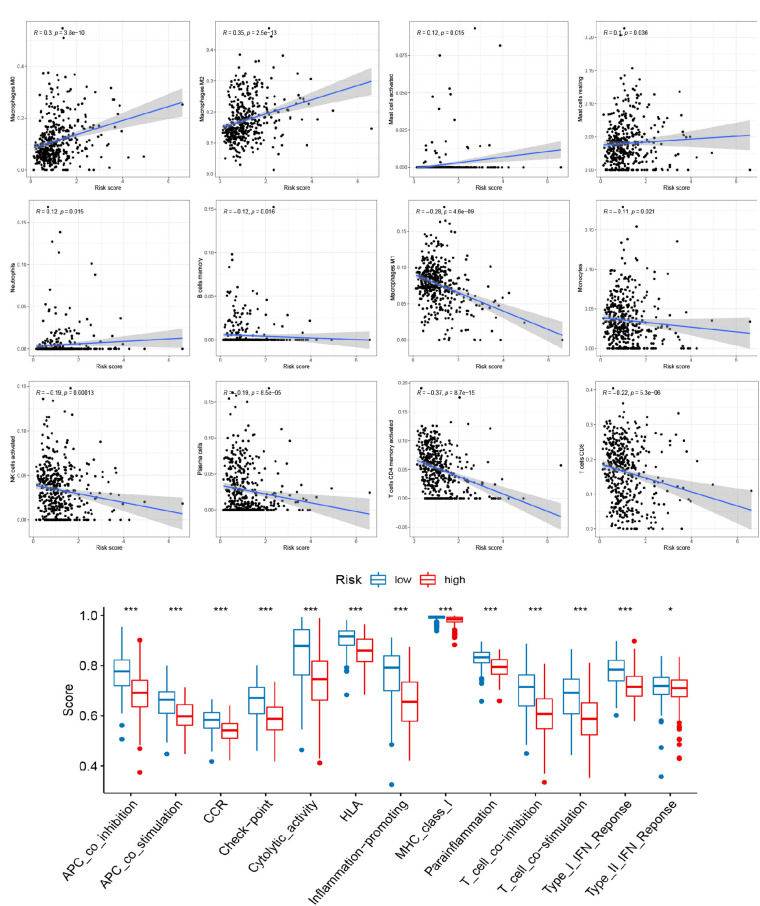
Relationships between the signature and tumor immune cell microenvironment. **(A)** Infiltration abundances of immune cell types. **(B)** 13 immune-related functions. *p < 0.05 and ***p < 0.001.

**FIGURE 5:**
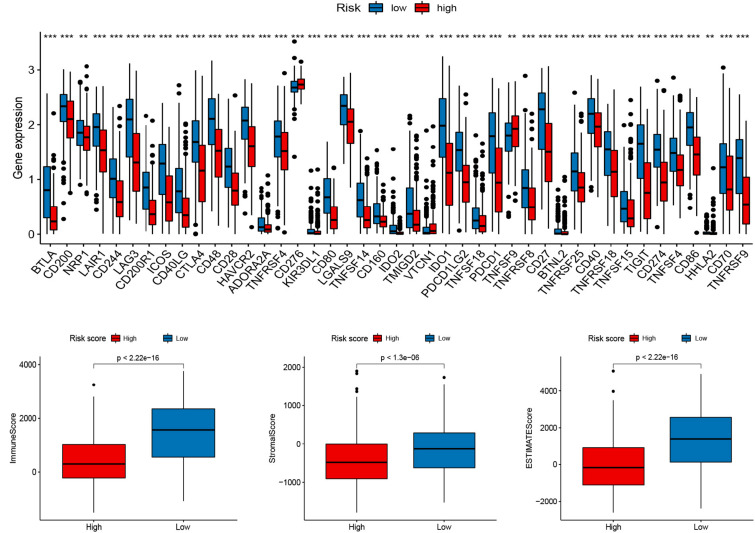
Relationships between the signature and immune checkpoints as well as TME score. **(A)** Immune checkpoints. **(B–D)** The TME score (Immune score, Stromal score, and Estimate score).

To explore the role of the signature on the TME of cutaneous melanoma, we analyzed the association between the signature, the abundance of 22 immune cells, 13 immune-related pathways, and TME score (Stromal score, Immune score, and Estimate score). Interestingly, high risk score was positively correlated with M0 macrophages, M2 macrophages, resting mast cells, activated mast cells, neutrophils, and negatively corrected with B cells memory, M1 macrophages, Monocytes, activated NK cells, Plasma cells, activated T cells CD4 memory, CD8 T cells (Figure 4A), and 13 immune-related pathways (Figure 4B). In addition, several vital immune-checkpoint-relevant genes were also analyzed and indicated that the risk score was significantly associated with the expression of the checkpoint markers, PD-1 (PDCD1), PD-L1 (CD274), and CTLA-4 (Figure 5A), implicating the potential roles of the signature model in the response to immunotherapy in cutaneous melanoma patients. Finally, the signature was negatively associated with immune score (*P* < 0.001), stromal score (*P* < 0.001), and ESTIMATE score (*P* < 0.001; Figures 5B–D).

### Drug Susceptibility Analysis

**FIGURE 6:**
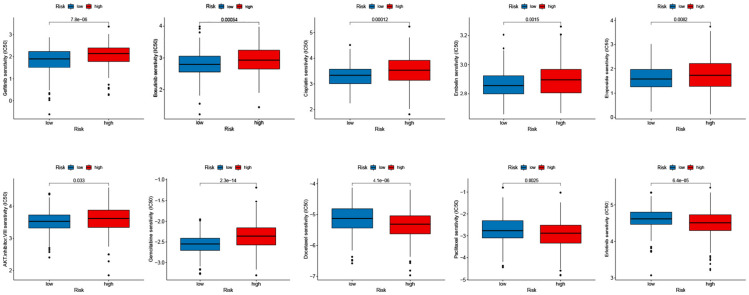
Relationships between the signature and drug susceptibility. **(A)** Gefitinib, Bosutinib, Cisplatin, Embelin, and Etoposide. **(B)** AKT inhibitor VIII, Gemcitabine, Docetaxel, Paclitaxel, and Erlotinib.

To manifest the application of antineoplastic drugs in melanoma patients hierarchically, we explored the antineoplastic drug susceptibility in the high- and low-risk groups based on the prognostic signature. As shown in Figure 6, after comprehensive analysis for the antineoplastic drugs, we noted that Gefitinib, Bosutinib, Cisplatin, Embelin, Etoposide, AKT inhibitor VIII, and Gemcitabine were more susceptible to the patients in the low-risk group compared with the patients in the high-risk group, while patients with high-risk seem more vulnerable to Docetaxel, Paclitaxel, and Erlotinib.

### Development of a Nomogram for Prognosis Prediction

**FIGURE 7:**
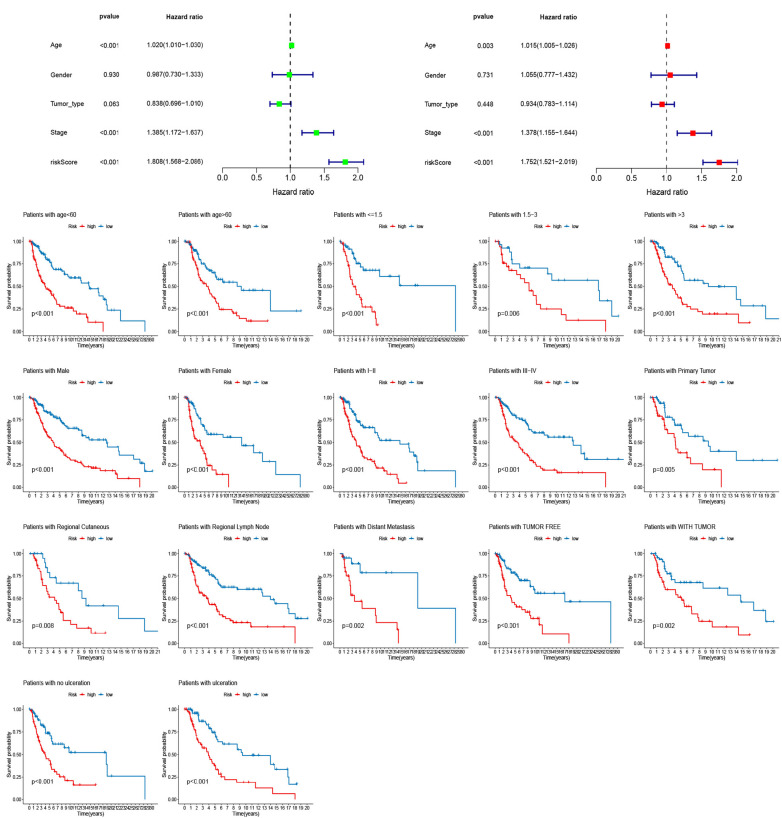
Univariate and multivariate Cox regression analyses assessing the independent prognostic value of risk score and clinical variables. **(A)** Univariate Cox regression analysis. **(B)** Multivariate Cox regression analysis. **(C)** The stratification analysis assessing predictive ability of the signature in different subgroups.

Univariate and multivariate Cox regression analyses were performed to determine whether the risk scores were independent risk factors of melanoma. The result confirmed that age, tumor stage, and risk score were independent prognostic factors (Figures 7A,B). To assess whether signature retained its prognostic value in various subgroups, we conducted a clinical stratification analysis. Kaplan-Meier analysis indicated that the OS of the high-risk score group was remarkably shorter than that of the low-risk score group (Figure 7C).

**FIGURE 8:**
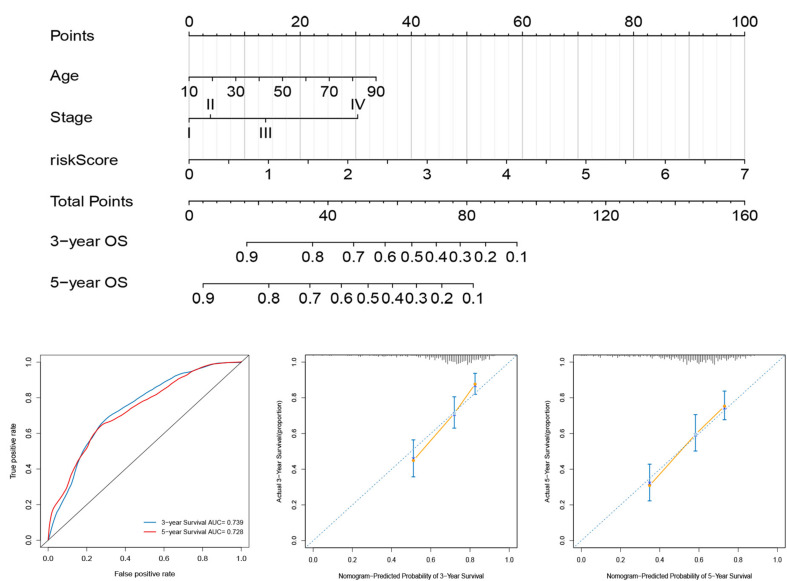
Construction and evaluation of a nomogram for survival prediction of melanoma patients based on risk score and clinical variables. **(A)** The nomogram was developed for predicting the 3- and 5-year OS of melanoma patients. **(B)** ROC curves for 3- and 5-year OS of the nomogram. **(C,D)** Calibration curves for predicting 3- and 5-year OS.

For the establishment of quantitative methods for cutaneous melanoma prognosis, a prognostic nomogram was established according to age, tumor stage, and risk score (Figure 8A). The AUCs of the nomogram at 3- and 5-year survival times were 0.739 and 0.728, respectively (Figure 8B). We used the calibration curve to show the prediction value of the nomogram, which results indicating that curve of the nomogram at 3, and 5 years OS were close to 45°line (Figures 8C,D), indicating high predictive accuracy.

### Gene Set Enrichment Analysis

**FIGURE 9:**
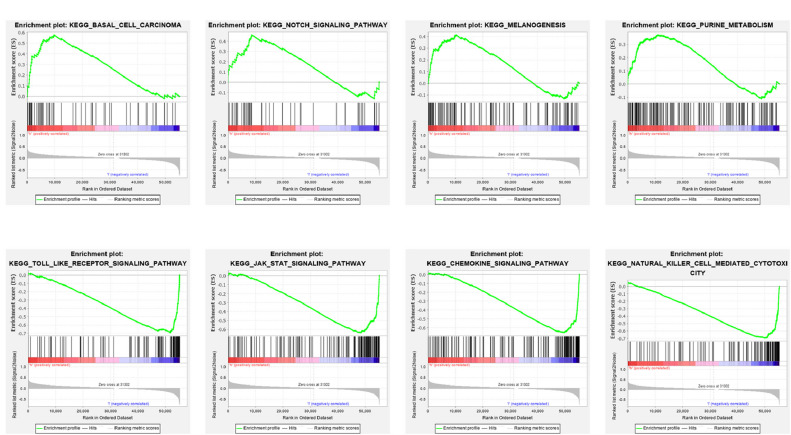
Gene set enrichment analysis (GSEA) for the signature based on RBPs. **(A)** Enriched KEGG pathways in the high-risk group. **(B)** Enriched KEGG pathways in the low-risk group.

Gene set enrichment analysis was used to discover the underlying biological mechanisms to further understand the development of cutaneous melanoma and the reasons for the different prognoses of patients with different scores. As shown in Figure 9, multiple significant signaling pathways were enriched in high- and low-risk group patients, but there was a different enrichment in the two groups. The high-risk group was mainly involved in basal cell carcinoma, notch signaling pathway, melanogenesis, and purine metabolism, while toll-like receptor, Jak-STAT, chemokine, natural killer cell-mediated cytotoxicity signaling pathway were the most significantly enriched signaling pathways in the low-risk group (Figure 9).

## Discussion

Despite breakthrough advancements in cutaneous melanoma treatment, some cutaneous melanoma patients still have a poor prognosis, especially when metastasis is detected. Due to the phenotype and genetic heterogeneity of malignant melanoma, conventional clinical features are still limited to accurately predict individual outcomes and survival. Accurate prognostic prediction and individualized clinical treatment strategy are the basis of precision medicine (Arnedos et al., 2014). Most of the established clinical markers for treatment response and prognosis of cutaneous melanoma are based on clinical features, and their accuracy and specificity are limited. Traditional AJCC TNM staging is mainly based on anatomical information and cannot adequately assess the prognosis of cutaneous melanoma patients. Therefore, exploring the molecular mechanisms and screening reliable melanoma-specific genomic signatures are urgently needed to improve prognosis assessment and individualized treatment. In recent years, with in-depth research on the regulatory role of RBPs in various RNA processes, researchers gradually realized the importance of RBPs in cancer. However, a systematic analysis of the relationship between RBPs and cutaneous melanoma is lacking. In the present study, we established an RBP-related prognostic signature, assessed the correlation between this model and prognosis as well as the immunotherapy response, and evaluated the potential clinical applications of the model.

The high-throughput “omics” data combined with bioinformatic analysis provided valid and economical methods to depict the prognostic value of RBPs in cutaneous melanoma. First, we combined the mRNA expression profiles of patients retrieved from the TCGA database and identified 1374 RBPs. Then, univariate, LASSO, and multivariate Cox regression analyses were carried out to develop a 7-RBP signature. The signature could classify patients into different risk subgroups with significantly different prognoses both in the TCGA and GEO sets. The reliability of the signature in predicting OS of melanoma patients was validated through ROC curves and PCA analyses between the two subgroups in the TCGA and GEO sets. To understand whether this newly identified RBP signature can specifically predict prognosis of cutaneous melanoma or has general prognostic value for other cancers, we evaluated prognostic value of the RBP signature in 32 cancer types using the TCGA pan-cancer data and found that the signature can also predict prognosis of other 5 types of cancer. Analyses of immune infiltrating, TME, immune checkpoint and drug susceptibility revealed significant differences between the two groups. The GSEA indicated that cancer-related processes and pathways were significantly enriched in the high-risk group defined by the RBP-related signature. Additionally, a nomogram combining 7-RBP-signature with clinical characteristics was performed to verify the robustness of the model for speculating OS in melanoma patients. The favorable predictive performance of the nomogram was validated by the discrimination and calibration curves.

The prognostic signature contained 7 RBPs. Some of the RBPs were found to affect the malignant phenotypes of tumors, such as RPP25, FBXO17, RBM43, and APOBEC3G. Consistent with the results of this study, previous studies have shown that RPP25 was significantly upregulated in tissues and cell lines of cervical cancer relative to the normal tissues. RPP25 can serve as a target gene of miR-3127-5p to promote the EMT process in cervical cancer (Yang et al., 2020). FBXO17, a negative regulator of glycogensynthase kinase-3β (GSK-3β), was identified by polyubiquitination and targeting of kinases to proteasomal degradation (Suber et al., 2017). FBXO17 was found to be upregulated in tumor tissues and promote malignant progression of cancer through different mechanisms, such as activation of Akt (Suber et al., 2018), or wnt/β-catenin pathway (Liu et al., 2019). Patients with elevated FBXO17 have a worse prognosis in multiple cancers, such as high-grade glioma (Du et al., 2018) and hepatocellular carcinoma (Liu et al., 2019). RNA-binding motif protein 43 (RBM43) was reported to be a tumor suppressor and correlated with poor prognosis in liver cancer. The overexpression of RBM43 can inhibit the proliferation of hepatocellular carcinoma cells, and decreased the growth of transplanted tumors *in vivo* through modulation of cyclin B1 expression (Feng et al., 2020). APOBEC3G has been reported to be dysregulated in tumor tissues and is associated with the prognosis of multiple cancers (Leonard et al., 2016; Han et al., 2020). There is an obvious correlation between APOBEC3G and tumor-infiltrating immune cells (Leonard et al., 2016; Han et al., 2020).

There is growing evidence that the TME, in which immune cells and molecules are important components, acts an important role in tumor development and the degree of immune cell infiltration is highly correlated with patient prognosis (Seager et al., 2017). The typical structure of the TME is composed of stromal components, endotheliocyte, mesenchymal stem cells, tumor-associated fibroblast and pericyte included, and immunocytes (Turley et al., 2015). With the recent development of technologies such as RNA-seq, it is possible to systematically analyze the TME and the functional diversity of tumor-infiltrating immune cells, the sensitivity of patients to immunotherapy, and the prognosis (Zhang and Zhang, 2019). Melanoma is one of the most immunogenic tumors because it has an incredibly high genomic mutation load and is most likely to trigger a specific adaptive anti-tumor immune response. Therefore, it has the greatest potential for response to immunotherapy (Marzagalli et al., 2019). In this research, we first explored the relationship between the RBP signature and tumor-infiltrating immune cells. We discovered that the relative contents of B cells memory, M1 macrophages, Monocytes, activated NK cells, Plasma cells, activated T cells CD4 memory, CD8 T cells, and 13 immune-related pathways were negatively correlated with the risk score. We further conducted correlational analysis for the signature and the expression of tumor immune checkpoint genes and noticed that with the risk score was significant with the expression of the checkpoint markers, such as PD-1, PD-L1, and CTLA-4, implicating the potential roles of the signature in the response to immunotherapy in cutaneous melanoma patients. Recently, the study of immune checkpoint therapy targeting PD-1 and CTLA-4 was blooming. The immunotherapies aiming at PD-1 and CTLA-4 have been widespread applied for melanoma (Specenier, 2016; Franklin et al., 2017). PD-1 is an important checkpoint receptor on the surface of T cells, and PD-1 combined with its agonist PD-L1 can also inhibit T cell activation. PD-L1 is expressed by melanoma cells or tumor-associated stroma, and this expression is closely related to the efficacy of anti-PD-1 immunotherapy (Taube et al., 2014). Several anti-PD-1 antibodies, such as nivolumab, ipilimumab, and pembrolizumab, have been approved for the treatment of melanoma (Franklin et al., 2017). Moreover, we further evaluated the association between the signature and TME. The result indicated that the signature was negatively associated with an immune score, estimate score, and ESTIMATE score might indicate that high-risk score inhibits immunoreaction to promote the progression of melanoma cells.

This study, for the first time, established a prognostic model based on RBPs, which could be a good tool for predicting the prognosis of cutaneous melanoma patients. Nevertheless, we have to admit that some limitations were also existing in our study. First, the results of this retrospective study based on bioinformatics analysis might exist a bit of bias, the prediction accuracy of the model needs to be further confirmed using prospective multicenter randomized controlled trials. The validation in cellular experiments, and animal and tissue models warrant further investigation. Second, the information from the TCGA database is limited and incomplete, which may reduce the predictive accuracy of the model.

## Data Availability Statement

The original contributions presented in the study are included in the article/Supplementary Material, further inquiries can be directed to the corresponding author/s.

## Author Contributions

JT and YZ made substantial contributions to the conception, design, interpretation, and preparation of the final manuscript. JT, CM, LY, and YS participated in the coordination of data acquisition and data analysis, and reviewed the manuscript. All authors contributed to the article and approved the submitted version.

## Conflict of Interest

The authors declare that the research was conducted in the absence of any commercial or financial relationships that could be construed as a potential conflict of interest.

## Publisher’s Note

All claims expressed in this article are solely those of the authors and do not necessarily represent those of their affiliated organizations, or those of the publisher, the editors and the reviewers. Any product that may be evaluated in this article, or claim that may be made by its manufacturer, is not guaranteed or endorsed by the publisher.

## Footnotes

1
https://portal.gdc.cancer.gov/

2
https://www.ncbi.nlm.nih.gov/geo/

3
https://cibersort.stanford.edu/

## Supplementary Material

Supplementary Figure 1 KM analysis of the OS between the high- and low-risk groups in 5 types of cancer using the TCGA pan-cancer data.

Supplementary Figure 2 KM analysis of the OS between the high- and low-risk groups in other 27 types of cancer using the TCGA pan-cancer data.

## Abbreviations

RBPs: RNA binding proteins
TCGA: The Cancer Genome Atlas
GEO: gene expression omnibus
LASSO: least absolute shrinkage and selection operator
TME: tumor microenvironment
OS: overall survival
GSEA: gene set enrichment analysis
AUC: area under curve
KM: Kaplan-Meier
PCA: principal component analysis
ROC: receiver operating characteristic
BLCA: bladder urothelial carcinoma
BRCA: breast invasive carcinoma
OV: ovarian serous cystadenocarcinoma
MESO: mesothelioma
LUAD: lung adenocarcinoma.

## References

[B1] Adolph D. Flach N. Mueller K. Ostareck D. H. Ostareck-Lederer A. (2007). Deciphering the cross talk between hnRNP K and c-Src: the c-Src activation domain in hnRNP K is distinct from a second interaction site. *Mole. Cell. Biol.* 27 1758–1770. 10.1128/mcb.02014-06 17178840 PMC1820454

[B2] Arnedos M. Soria J. C. Andre F. Tursz T. (2014). Personalized treatments of cancer patients: a reality in daily practice, a costly dream or a shared vision of the future from the oncology community? *Cancer Treat. Rev.* 40 1192–1198. 10.1016/j.ctrv.2014.07.002 25441102

[B3] Baltz A. G. Munschauer M. Schwanhäusser B. Vasile A. Murakawa Y. Schueler M. (2012). The mRNA-bound proteome and its global occupancy profile on protein-coding transcripts. *Mol. Cell* 46 674–690. 10.1016/j.molcel.2012.05.021 22681889

[B4] Castello A. Fischer B. Eichelbaum K. Horos R. Beckmann B. M. Strein C. (2012). Insights into RNA biology from an atlas of mammalian mRNA-binding proteins. *Cell* 149 1393–1406. 10.1016/j.cell.2012.04.031 22658674

[B5] Corley M. Burns M. C. Yeo G. W. (2020). How RNA-Binding Proteins Interact with RNA: Molecules and Mechanisms. *Mol. Cell* 78 9–29. 10.1016/j.molcel.2020.03.011 32243832 PMC7202378

[B6] Cunningham F. Amode M. R. Barrell D. Beal K. Billis K. Brent S. (2015). Ensembl 2015. *Nucleic Acids Res.* 43 D662–D669. 10.1093/nar/gku1010 25352552 PMC4383879

[B7] Diamantopoulos P. Gogas H. (2016). Melanoma immunotherapy dominates the field. *Ann. Transl. Med.* 4:269. 10.21037/atm.2016.06.32 27563656 PMC4971379

[B8] Du D. Yuan J. Ma W. Ning J. Weinstein J. N. Yuan X. (2018). Clinical significance of FBXO17 gene expression in high-grade glioma. *BMC Cancer* 18:773. 10.1186/s12885-018-4680-3 30064493 PMC6069786

[B9] Feng H. Liu J. Qiu Y. Liu Y. Saiyin H. Liang X. (2020). RNA-binding motif protein 43 (RBM43) suppresses hepatocellular carcinoma progression through modulation of cyclin B1 expression. *Oncogene* 39 5495–5506. 10.1038/s41388-020-1380-7 32632220

[B10] Franklin C. Livingstone E. Roesch A. Schilling B. Schadendorf D. (2017). Immunotherapy in melanoma: Recent advances and future directions. *Eur. J. Surg. Oncol.* 43 604–611. 10.1016/j.ejso.2016.07.145 27769635

[B11] Gallardo M. Malaney P. Aitken M. J. L. Zhang X. Link T. M. Shah V. (2020). Uncovering the Role of RNA-Binding Protein hnRNP K in B-Cell Lymphomas. *J. Natl. Cancer Inst.* 112 95–106. 10.1093/jnci/djz078 31077320 PMC7489062

[B12] Han W. Xu J. Shen G. L. (2020). Prognostic implication and functional annotations of APOBEC3G expression in patients with Melanoma. *J. Cancer* 11 5245–5256. 10.7150/jca.46383 32742470 PMC7378923

[B13] Hyams D. M. Cook R. W. Buzaid A. C. (2019). Identification of risk in cutaneous melanoma patients: Prognostic and predictive markers. *J. Surg. Oncol.* 119 175–186. 10.1002/jso.25319 30548543 PMC6590387

[B14] Kwon S. C. Yi H. Eichelbaum K. Föhr S. Fischer B. You K. T. (2013). The RNA-binding protein repertoire of embryonic stem cells. *Nat. Struct. Mol. Biol.* 20 1122–1130. 10.1038/nsmb.2638 23912277

[B15] Leonard B. Starrett G. J. Maurer M. J. Oberg A. L. Van Bockstal M. Van Dorpe J. (2016). APOBEC3G Expression Correlates with T-Cell Infiltration and Improved Clinical Outcomes in High-grade Serous Ovarian Carcinoma. *Clin. Cancer Res.* 22 4746–4755. 10.1158/1078-0432.Ccr-15-2910 27016308 PMC5026552

[B16] Liu F. H. Cui Y. P. He Y. K. Shu R. H. (2019). FBXO17 promotes malignant progression of hepatocellular carcinoma by activating wnt/β-catenin pathway. *Eur. Rev. Med. Pharm. Sci.* 23 8265–8273. 10.26355/eurrev_201910_19137 31646557

[B17] Marzagalli M. Ebelt N. D. Manuel E. R. (2019). Unraveling the crosstalk between melanoma and immune cells in the tumor microenvironment. *Semin. Cancer Biol.* 59 236–250. 10.1016/j.semcancer.2019.08.002 31404607

[B18] Masuda K. Kuwano Y. (2019). Diverse roles of RNA-binding proteins in cancer traits and their implications in gastrointestinal cancers. *Wiley Interdiscip. Rev. RNA.* 10:e1520. 10.1002/wrna.1520 30479000

[B19] Mohibi S. Chen X. Zhang J. (2019). Cancer the’RBP’eutics-RNA-binding proteins as therapeutic targets for cancer. *Pharmacol. Ther.* 203:107390. 10.1016/j.pharmthera.2019.07.001 31302171 PMC6848768

[B20] Newman A. M. Liu C. L. Green M. R. Gentles A. J. Feng W. Xu Y. (2015). Robust enumeration of cell subsets from tissue expression profiles. *Nat. Methods* 12 453–457. 10.1038/nmeth.3337 25822800 PMC4739640

[B21] Schadendorf D. Hauschild A. (2014). Melanoma in 2013: Melanoma–the run of success continues. *Nat. Rev. Clin. Oncol.* 11 75–76. 10.1038/nrclinonc.2013.246 24419300

[B22] Schuschel K. Helwig M. Hüttelmaier S. Heckl D. Klusmann J. H. Hoell J. I. (2020). RNA-Binding Proteins in Acute Leukemias. *Int. J. Mol. Sci.* 21:21103409. 10.3390/ijms21103409 32408494 PMC7279408

[B23] Seager R. J. Hajal C. Spill F. Kamm R. D. Zaman M. H. (2017). Dynamic interplay between tumour, stroma and immune system can drive or prevent tumour progression. *Converg. Sci. Phys. Oncol.* 3:86. 10.1088/2057-1739/aa7e86 30079253 PMC6070160

[B24] Shi H. Li H. Yuan R. Guan W. Zhang X. Zhang S. (2018). PCBP1 depletion promotes tumorigenesis through attenuation of p27(Kip1) mRNA stability and translation. *J. Exp. Clin. Cancer Res.* 37:187. 10.1186/s13046-018-0840-1 30086790 PMC6081911

[B25] Siegel R. L. Miller K. D. Jemal A. (2020). Cancer statistics, 2020. *CA Cancer J. Clin.* 70 7–30. 10.3322/caac.21590 31912902

[B26] Specenier P. (2016). Ipilimumab in melanoma. *Expert Rev. Anticancer Ther.* 16 811–826. 10.1080/14737140.2016.1211936 27403706

[B27] Suber T. Wei J. Jacko A. M. Nikolli I. Zhao Y. Zhao J. (2017). SCF(FBXO17) E3 ligase modulates inflammation by regulating proteasomal degradation of glycogen synthase kinase-3β in lung epithelia. *J. Biol. Chem.* 292 7452–7461. 10.1074/jbc.M116.771667 28298444 PMC5418045

[B28] Suber T. L. Nikolli I. O’Brien M. E. Londino J. Zhao J. Chen K. (2018). FBXO17 promotes cell proliferation through activation of Akt in lung adenocarcinoma cells. *Respir. Res.* 19:206. 10.1186/s12931-018-0910-0 30359271 PMC6203195

[B29] Swetter S. M. Tsao H. Bichakjian C. K. Curiel-Lewandrowski C. Elder D. E. Gershenwald J. E. (2019). Guidelines of care for the management of primary cutaneous melanoma. *J. Am. Acad. Dermatol.* 80 208–250. 10.1016/j.jaad.2018.08.055 30392755

[B30] Tang D. Y. Ellis R. A. Lovat P. E. (2016). Prognostic Impact of Autophagy Biomarkers for Cutaneous Melanoma. *Front. Oncol.* 6:236. 10.3389/fonc.2016.00236 27882308 PMC5101199

[B31] Taube J. M. Klein A. Brahmer J. R. Xu H. Pan X. Kim J. H. (2014). Association of PD-1, PD-1 ligands, and other features of the tumor immune microenvironment with response to anti-PD-1 therapy. *Clin. Cancer Res.* 20 5064–5074. 10.1158/1078-0432.Ccr-13-3271 24714771 PMC4185001

[B32] Turley S. J. Cremasco V. Astarita J. L. (2015). Immunological hallmarks of stromal cells in the tumour microenvironment. *Nat. Rev. Immunol.* 15 669–682. 10.1038/nri3902 26471778

[B33] Wang X. Guo J. Che X. Jia R. (2019). PCBP1 inhibits the expression of oncogenic STAT3 isoform by targeting alternative splicing of STAT3 exon 23. *Int. J. Biol. Sci.* 15 1177–1186. 10.7150/ijbs.33103 31223278 PMC6567812

[B34] Weiße J. Rosemann J. Krauspe V. Kappler M. Eckert A. W. Haemmerle M. (2020). RNA-Binding Proteins as Regulators of Migration, Invasion and Metastasis in Oral Squamous Cell Carcinoma. *Int. J. Mol. Sci.* 21:21186835. 10.3390/ijms21186835 32957697 PMC7555251

[B35] Yang J. Hou S. Liang B. (2020). LINC00319 promotes migration, invasion and epithelial-mesenchymal transition process in cervical cancer by regulating miR-3127-5p/RPP25 axis. *Vitro Cell. Dev. Biol. Anim.* 56 145–153. 10.1007/s11626-019-00425-5 31942724

[B36] Yoshihara K. Shahmoradgoli M. Martínez E. Vegesna R. Kim H. Torres-Garcia W. (2013). Inferring tumour purity and stromal and immune cell admixture from expression data. *Nat. Commun.* 4:2612. 10.1038/ncomms3612 24113773 PMC3826632

[B37] Zhang J. Cho S. J. Shu L. Yan W. Guerrero T. Kent M. (2011). Translational repression of p53 by RNPC1, a p53 target overexpressed in lymphomas. *Genes Dev.* 25 1528–1543. 10.1101/gad.2069311 21764855 PMC3143942

[B38] Zhang J. Xu E. Ren C. Yan W. Zhang M. Chen M. (2014). Mice deficient in Rbm38, a target of the p53 family, are susceptible to accelerated aging and spontaneous tumors. *Proc. Natl. Acad. Sci. U S A.* 111 18637–18642. 10.1073/pnas.1415607112 25512531 PMC4284600

[B39] Zhang L. Zhang Z. (2019). Recharacterizing Tumor-Infiltrating Lymphocytes by Single-Cell RNA Sequencing. *Cancer Immunol. Res.* 7 1040–1046. 10.1158/2326-6066.Cir-18-0658 31262773

[B40] Zhang M. Zhang Y. Xu E. Mohibi S. de Anda D. M. Jiang Y. (2018). Rbm24, a target of p53, is necessary for proper expression of p53 and heart development. *Cell Death Diff.* 25 1118–1130. 10.1038/s41418-017-0029-8 29358667 PMC5988652

[B41] Zhang X. Di C. Chen Y. Wang J. Su R. Huang G. (2020). Multilevel regulation and molecular mechanism of poly (rC)-binding protein 1 in cancer. *Faseb J.* 34 15647–15658. 10.1096/fj.202000911R 33058239

[B42] Zhang Y. Meng L. Xiao L. Liu R. Li Z. Wang Y. L. (2018). The RNA-Binding Protein PCBP1 Functions as a Tumor Suppressor in Prostate Cancer by Inhibiting Mitogen Activated Protein Kinase 1. *Cell. Physiol. Biochem.* 48 1747–1754. 10.1159/000492315 30078000

