# Supplementary figures and images for: Prognostic Value and Immunological Characteristics of a Novel RNA Binding Protein Signature in Cutaneous Melanoma
Source: Front Genet. 2021 Aug 31;12:723796. doi: 10.3389/fgene.2021.723796 (PMC8438157; doi:10.3389/fgene.2021.723796)

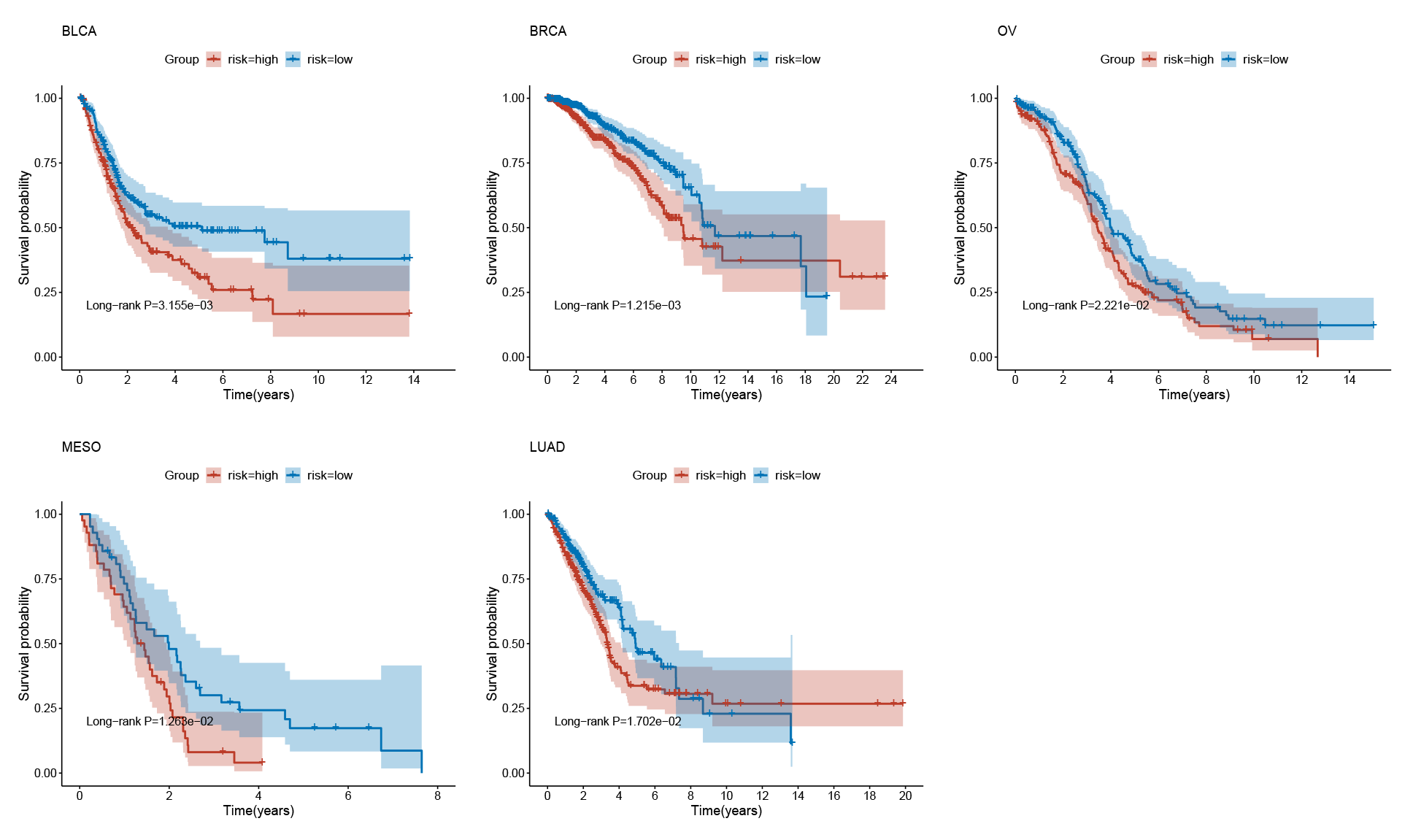

Supplement: Supplementary Figure 1 — KM analysis of the OS between the high- and low-risk groups in 5 types of cancer using the TCGA pan-cancer data. [file Image_1.TIF]

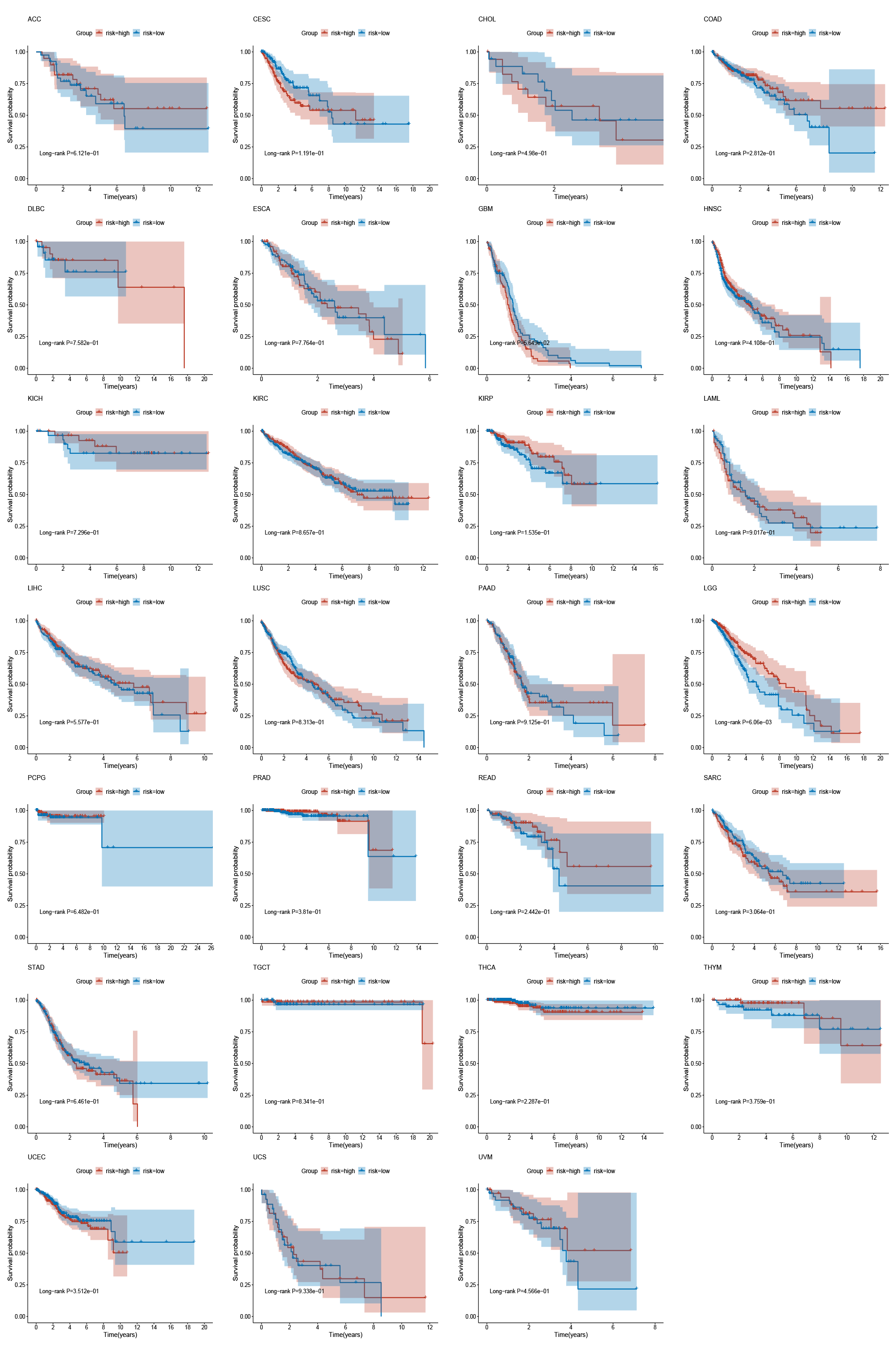

Supplement: Supplementary Figure 2 — KM analysis of the OS between the high- and low-risk groups in other 27 types of cancer using the TCGA pan-cancer data. [file Image_2.TIF]
